# The complete chloroplast genome of *Pyrrosia bonii* (Polypodiaceae), an important ornamental and medical fern

**DOI:** 10.1080/23802359.2018.1491347

**Published:** 2018-08-13

**Authors:** Shicheng Cai, Xiang Cai, Shufeng Li, Shanshan Liu, Zhen Wang, Ting Wang, Yingjuan Su

**Affiliations:** a School of Life Sciences, Sun Yat-sen University, Guangzhou, China;; b College of Life Sciences, Nanjing Agricultural University, Nanjing, China;; c College of Life Sciences, South China Agricultural University, Guangzhou, China;; d Research Institute of Sun Yat-sen University in Shenzhen, Shenzhen, China

**Keywords:** *Pyrrosia bonii*, chloroplast genome, ornamental and medical fern, phylogenetic analysis

## Abstract

*Pyrrosia bonii* is an important ornamental and medical fern from Polypodiaceae. Its complete chloroplast genome sequence was characterized through *de novo* assembly with Illumina sequencing data. The genome size is 158,174 bp, with large single copy (LSC, 82,479 bp) and small single copy (SSC, 21,723 bp) regions separated by a pair of inverted repeats (IR, 26,986 bp). A total of 132 genes were identified, including 88 protein coding genes, 35 tRNA genes, eight rRNA genes, and one pseudogene. Maximum-likelihood phylogenetic tree revealed that *P. bonii* was most closely related to *Lepisorus clathratus*. The study will greatly facilitate the chloroplast phylogenomics of Polypodiaceae.

*Pyrrosia bonii* (Christ ex Giesenh.) Ching is an epiphytic tropical fern belonging to Polypodiaceae. It has two key characters of this genus, i.e. stellate hairs and connective venation pattern (Zhang et al. 2013). As a terrestrial fern, the species mainly grows on rocks in forest at an altitude of 300–1100 m with distribution in China including Guangxi and Guizhou, as well as Vietnam. *Pyrrosia bonii* has not only unique ornamental value, but also high medical value. The dried leaves of *P. bonii* have been used to substitute for medicinal materials ‘Shiwei’ to treat urinary infection and urolithiasis, although it has not been recorded in the Chinese pharmacopoeia (National Pharmacopoeia Committee 2010). In addition, there are many difficulties and controversies in species-level classification in *Pyrrosia* due to high similarity in morphology (Wei et al. 2017). The monophyly and systematic position of *Pyrrosia* in Platycerioideae also occur controversy (Hovenkamp 1986). Hence, sequencing of complete chloroplast genome of *P. bonii* will be conducive to a deeper and better understanding of classification and phylogeny of *Pyrrosia.*

Total genomic DNA was extracted from the fresh leaves of *P. bonii* using the Tiangen Plant Genomic DNA Kit (Tiangen Biotech Co., Beijing, China), which were collected from South China Botanical Garden, Chinese Academy of Sciences (23°11′3.56″N, 113°21′43.28″E). The specimen was saved in Herbarium of Sun Yat-sen University (SYS; voucher: *SS Liu 201615*). After DNA was broken by Covaris M220 (Covaris Inc., Woburn, MS), an average 300 bp Illumina library was constructed and sequenced on the Illumina Hiseq 2500 platform (Illumina, San Diego, CA). We separately used Trimmomatic v0.32 (Bolger et al. 2014) and FastQC v0.10.0 (Andrews 2010) to trim reads and visualize the quality of the clean reads. A total of 1.86 G clean data was further assembled into the complete chloroplast genome using Velvet v1.2.07 (Zerbino and Birney 2008), which was annotated through DOGMA (Wyman et al. 2004) and tRNAscan-SE (Schattner et al. 2005). After we aligned complete chloroplast genome sequences of 11 ferns and *Alsophila spinulosa* as an outgroup using MAFFT v7.394 (Kazutaka and Standley 2013), phylogenetic relationships were presented by RAxML v8.2.10 (Stamatakis 2014) based on the maximum-likelihood method with 1000 bootstrap replicates.

**Figure 1.**
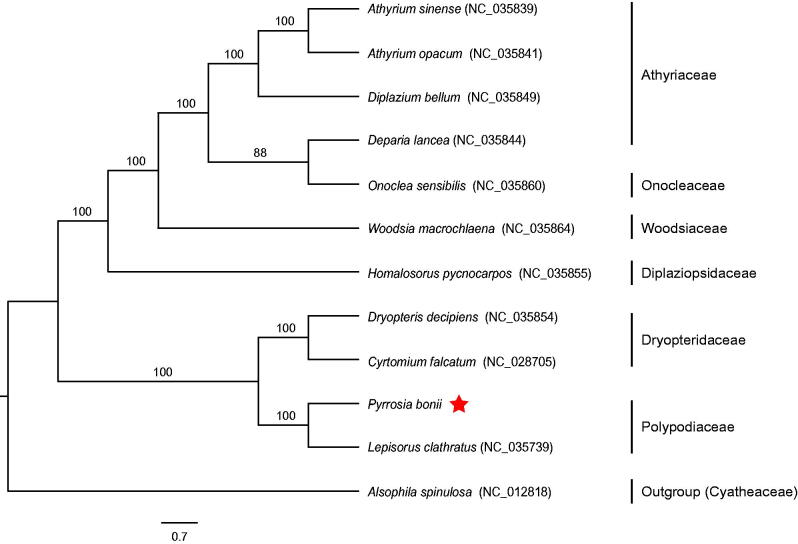
Using RAxML v8.2.10, ML phylogenetic tree was constructed based on complete chloroplast genome sequences of 11 ferns and Alsophila s*pinulosa* as an outgroup. The numbers in the nodes indicated the support values with 1000 bootstrap replicates.

The complete chloroplast genome of *P. bonii* (GenBank accession no. MH352390) is a circular DNA molecule of 158,174 bp in length with a typical quadripartite structure including a large single copy (LSC) region (82,479 bp), a small single copy (SSC) region (21,723 bp) and a pair of inverted repeats (IRs) regions (26,986 bp). We annotated 132 genes, consisting of 88 protein coding genes (PCGs), 35 tRNA genes, eight rRNA genes and one pseudogene (*ndh*B). Among these genes, 15 genes (*ndhB, rps16, atpF, rpoC1, petB, petD, ndhA, rpl16, rpl2, trnG-UCC, trnV-UAC, trnA-UGC, trnI-GAU, trnL-UAA,* and *trnT-UGU*) contain one intron, while three genes (*ycf3, clpP,* and *rps12*) possess two introns. Fourteen genes are duplicated in two IR regions, involving in four PCGs (*rps12, rps7, psbA,* and *ycf2*), six tRNA genes (*trnN-GUU, trnH-GUG, trnI-GAU, trnA-UGC, trnT-UGU,* and *trnR-ACG*), and four rRNA genes (*rrn4.5, rrn5, rrn16,* and *rrn23*). The overall GC contents of chloroplast genome are 41.6%. ML tree reveals that *P. bonii* was most closely related to *Lepisorus clathratus* with 100% support value (Figure 1). Our study will greatly facilitate the chloroplast phylogenomics of Polypodiaceae.
